# Optimizing in vitro nutrient and ex vitro soil mediums-driven responses for multiplication, rooting, and acclimatization of pineapple

**DOI:** 10.1038/s41598-023-28359-9

**Published:** 2023-01-23

**Authors:** Maqsood Ahmed Lakho, Mushtaque Ahmed Jatoi, Najamuddin Solangi, Adel Ahmed Abul-Soad, Muneer Ahmed Qazi, Gholamreza Abdi

**Affiliations:** 1 Department of Botany/Date Palm Research Institute, Shah Abdul Latif University, Khairpur, Sindh Pakistan; 2 Tropical Fruit Department, Horticulture Research Institute, Agricultural Research Center, Cairo, Egypt; 3 Institute of Microbiology, Shah Abdul Latif University, Khairpur, Sindh Pakistan; 4 Department of Biotechnology, Persian Gulf Research Institute, Persian Gulf University, Bushehr, 75169 Iran

**Keywords:** Biotechnology, Plant sciences

## Abstract

A comprehensive study was carried out on in vitro multiplication and rooting using the medium enriched with different plant growth regulators and acclimatization of pineapple cv. ‘Smooth Cayenne’ using different soil growing substrates. The significantly highest shoot buds (Avg. 16.7) were obtained on the medium comprising 2.0 mg L^−1^ BA (6-Benzylaminopurine). Results showed that 1.0 mg L^−1^ IBA (Indole-3-butyric acid) increased the thickness and length of white adventitious roots and resulted in a significantly highest number of roots (Avg. 8) and root length (6.15 cm). Plantlets with healthy, multiple roots were transplanted in several soil combinations of river silt, bolhari (yellow sand), and peat moss. However, the significantly highest survival (100%) of plantlets in the greenhouse was obtained on the soil medium containing only peat moss. Furthermore, soil mixtures of bolhari and peat moss (1:1) and river silt alone exhibited 98.9% and 95.1% survivability of plantlets, which was also considered equally significant (at 5% probability level). The in vitro and ex vitro protocols optimized in the current study can be applied commercially for pineapple crop worldwide.

## Introduction

Pineapple (*Ananas comosus* L. Merr.) is an important edible horticultural crop of *Bromeliaceae* cultivated in tropical and subtropical areas^1,2^. It accounts for nearly 20% of global tropical fruit production, after mangoes and bananas. The world production of pineapple is 27.81 million tons with 1.07 million ha of cultivation^3^. The top three pineapple producers worldwide are the Philippines, Costa Rica, and Brazil. Pineapple is cultivated extensively for its sweet, juicy fruit, often exported fresh or in cans. It has a high fresh market value due to the source of vitamins (A, B, C), calcium, iron, and the enzyme bromelain^4^. Vegetative propagules such as slips, suckers, crowns, and ratoons are used to propagate pineapple^5^. Traditional pineapple propagation is occasionally hindered by a lack of quality and a poor rate of sucker formation, as well as diseases that result in significant production losses^6,7^. Large-scale propagation of pineapple plantlets via in vitro methods offers an excellent opportunity for the growers^7^. Different reports are available discussing the establishment of micropropagation protocols for pineapple^8–13^. The PGRs are generally used for in vitro propagation and rooting in pineapple plants. Different auxins such as 1-Naphthaleneacetic acid (NAA), Indole-3-acetic acid (IAA), Indole-3-butyric acid (IBA) were previously employed for root formation in many plant species^14,15^. In vitro rooting is usually done with NAA and IBA, used alone or in combination^16^. Teng^17^, employed higher amounts of NAA and BA to induce in vitro rooting in pineapple. Furthermore, Danso et al.^18^ used a combination of NAA and IBA to improve pineapple rooting. The current study also focused on the application of BA in the medium for getting high proliferation rate of little shoot buds via in vitro growth stage. BA was found to be more effective than other cytokinin for multiplication and shoot development^19^. In brief, BA is involved in the multiplication of shoots at the initial stage. However, very high doses of BA were not applied.

The plants grown in vitro, with limited gas exchange, high moisture, low light, and a sucrose-based medium that may hinder photosynthesis, making difficult acclimatization of micropropagated plantlets and causing substantial losses^20^. Since two environments (in vitro and ex vitro) have different physiological conditions, successful acclimatization is considered a vital stage after in vitro stage. The selection of an appropriate soil media, on the other hand, decreases plant mortality throughout the acclimatization phase. A suitable substrate should be hard and dense to obtain good roots. The substrate should be devoid of weeds, nematodes, diseases, and high salt levels. Mixtures of soil/peat and soil/sand/humus resulted in better development of micropropagated pineapple plantlets^21^. To achieve a 100% survival rate of tissue culture-derived pineapple plantlets, Farahani and Farahani^22^ utilized a mixture of garden soil, perlite, and compost (2:1:2). The already conducted experiments exhibit that age of shoots has significant effects on rooting responses during in vitro growth^15^. The size plays an important role in ex vitro growth^8^. However, the impacts of PGRs treatments on rooting and height of plantlets are rarely reported^23^, and the effect of shoot age ignored entirely.

The current study hence aimed to optimize in vitro multiplication, shoot and root development in response to different PGR-enriched mediums. The current study also examined how different soil mixtures affect pineapple plantlets' acclimatization in the greenhouse.

## Materials and methods

### Plant material

**Figure 1:**
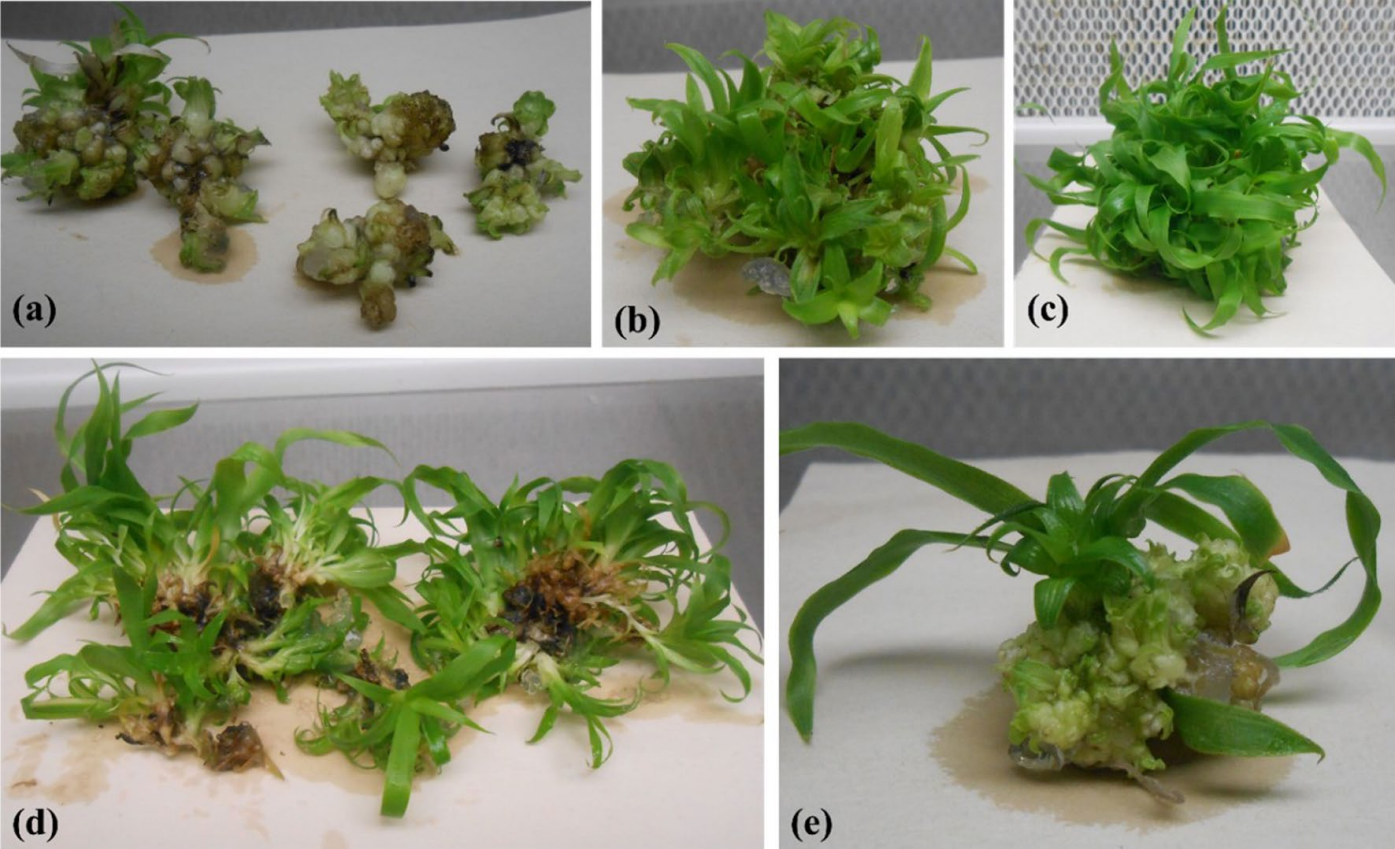
(**a**) Clusters of shoot buds, (**b**) Shoots emergence in the bud cluster, (**c**) Complete shoots formation in the bud cluster, (**d**) Isolation of shoots, (**e**) Formation of shoot bud cluster at the base of a plantlet.

The small bud clusters (Fig. 1a) were used as primary cultures, initially produced from meristematic (immature cells with the power of division) shoot tips (source of vegetative tissue for getting true-to-type plantlets) in Horticultural Research Institute, Agricultural Research Centre, Cairo, Egypt and transported to the biotechnology laboratory of Date Palm Research Institute, Shah Abdul Latif University, Khairpur by preserving in sealed glass vessels and cooled sterile environment ensuring no entry of any pathogens inside culture vessel as per National guidelines and legislations. The in vitro grown plantlets (10–12 cm long) were prepared from shoot buds and cultured on different treatments of PGRs for excellent shoot formation, elongation, and rooting.

### Effect of BA concentrations on in vitro multiplication of pineapple

The Murashige and Skoog^24^ medium comprising 0.0, 0.5, 1.0, 2.0 mg L^−1^ BA, 30 g L^−1^ sucrose solidified with 6 g L^−1^ agar used to multiply shoot bud cultures to produce small plantlets (avg. 7 cm long).

### Effect of different auxin types and concentrations on in vitro rooting of pineapple

To investigate the efficacy of auxins in inducing roots in pineapple plantlets, the MS medium^24^ solidified with 6 g L^−1^ agar containing different auxins (IBA, NAA, IAA) at three different treatments (0.1, 1.0, 3.0 mg L^−1^) along with three control replicates in addition to 30 g L^−1^ sucrose was used.

### Cultural conditions and data collection

The plantlets were incubated in a growth room at 25 ± 2 °C under 16 h of photoperiod every day to develop root and shoot. Subculturing was carried out after every 1-month interval. The data regarding root number, root length, leaf number, leaf length and width were taken after sub-culturing.

### Effect of different soil mixtures on acclimatization of plantlets

Plantlets taken out of jars with complete care, avoiding any damage to the soft stem, leaves, and roots, followed by rinsing in sterilized cooled water to clean the gel attached to roots and leaves. Later the plantlets were immersed in a 0.5% fungicide solution (Copper Oxychloride, Syngenta) for five min and transplanted into 250 mm plastic pots with varying soil mixtures, including bolhari (yellow sand), river silt, peat moss, and peat moss + bolhari (1:1 v/v). Plantlets were then kept for one week in a glasshouse under humidity (90–95%) and sunlight, covered with a transparent polyethene sheet. The plantlets were watered regularly and sprayed with fungicide at different intervals. After one month of transplanting, the survival percentage of plantlets was calculated, and leaf length (cm), leaf width, and leaf number were noted every month for up to three months.

### Statistical analysis

The ANOVA and LSD analyses were performed with Statistix (Version 8.1). The mean, standard deviation, and standard error of all experimental data collected from three replicates were calculated using Microsoft Excel 2016. The separation of means among treatments was assessed using the LSD test at 5%.

## Results and discussion

### Effect of different treatments of BA on shoot bud multiplication

This experiment aimed to optimize the best treatment for the high-frequency proliferation of small shoot buds (Fig. 1a) and the conversion of these buds into plantlets. The addition of BA in the multiplication medium at 2.0 mg L^−1^ induced the highest number of shoots (Fig. 1b) and shoot bud cluster (avg. 5 g) at the base of each plantlet (Fig. 1e) in two months. Data presented in Table 1 indicate that the significantly highest shoot bud multiplication and formation of healthy shoots (avg. 16.7 shoots from 5 g bud cluster) were obtained on a medium comprising of 2.0 mg L^−1^ BA (Fig. 1d). Significantly lowest formation of shoots (avg. 3 shoots) was noted on the medium without any PGR and the medium containing 0.5 mg L^−1^ BA (avg. 7.3 shoots were formed). Ibrahim et al.^10^ also noted the least response on the lower treatment of BA (0.5 mg L^−1^). In addition to small shoots, shoot buds further multiplied at the base of these shoots on the same medium within two months (Fig. 1e). Moreover, the production frequency of adventive shoot buds at the base of individual plantlets was significantly highest (Fig. 1e) as compared to the base of plantlets in the cluster (Fig. 1d) which resulted in the least production of shoot buds at the basal side of plantlets. The reason behind a smaller number of shoot bud formations may be due to the dense growth of plantlets which compete for nutrient and hormone requirements in the artificial growth medium.

**Table 1:** Effect of different treatments of BA on the formation of plantlets from small bud clusters under light (16 h photoperiod).

BA (mg L^−1^)	Plantlet formation/5 g bud cluster
0.0	3.0^d^
0.5	7.3^c^
1.0	10.5^b^
2.0	16.7^a^
LSD at 0.05%	0.005

Further, it was noted that, surprisingly no plantlet underwent shoot tip necrosis for up to two months on the same medium. However, if the subculture period was increased to more than two months, it led to necrosis in the tip of leaves due to the high proliferation of buds on the base of the plantlet on the medium comprising 2.0 mg L^−1^ BA. Besides the high-frequency proliferation rate and formation of new plantlets, none of the older leaves died in two months of growth (Fig. 1c). Abul-Soad et al.^25^ obtained significant results in getting the maximum number of shoots by enhancing BA to 2.0 mg L^−1^ within 2 months of culture. Other studies used full-strength MS medium with varying BA treatments for in vitro production of pineapple from pineapple crown explants^4,7^. Al-Saif et al.^26^ obtained the maximum number of long shoots of pineapple from terminal bud culture on the MS medium comprising 2.0 mg L^−1^ BA. The current study examined the effects of different BA concentrations on adventitious bud proliferation and the development of these buds into shoots. Different levels of BA have been compared in various plant species for shoot development and multiplication. For example, adding 1 mg L^−1^ BA to MS media increased *Amygdalus communis* multiplication rate^19^. Borthakur et al.^27^ found that adding 0.75 mg L^−1^ BAP to media increased the number of shoots of *Albizzia odoratissima*, with an average of 10 shoots per explant. Further noted that high-frequency bud multiplication (Fig. 1e) was acquired on the medium comprising 2.0 g L^−1^ BA than the rest of the BA treatments added in the medium and the medium lacking PGRs.

### Effect of different treatments of auxins (IBA, NAA, IAA) on in vitro rooting of pineapple plantlets

**Figure 2:**
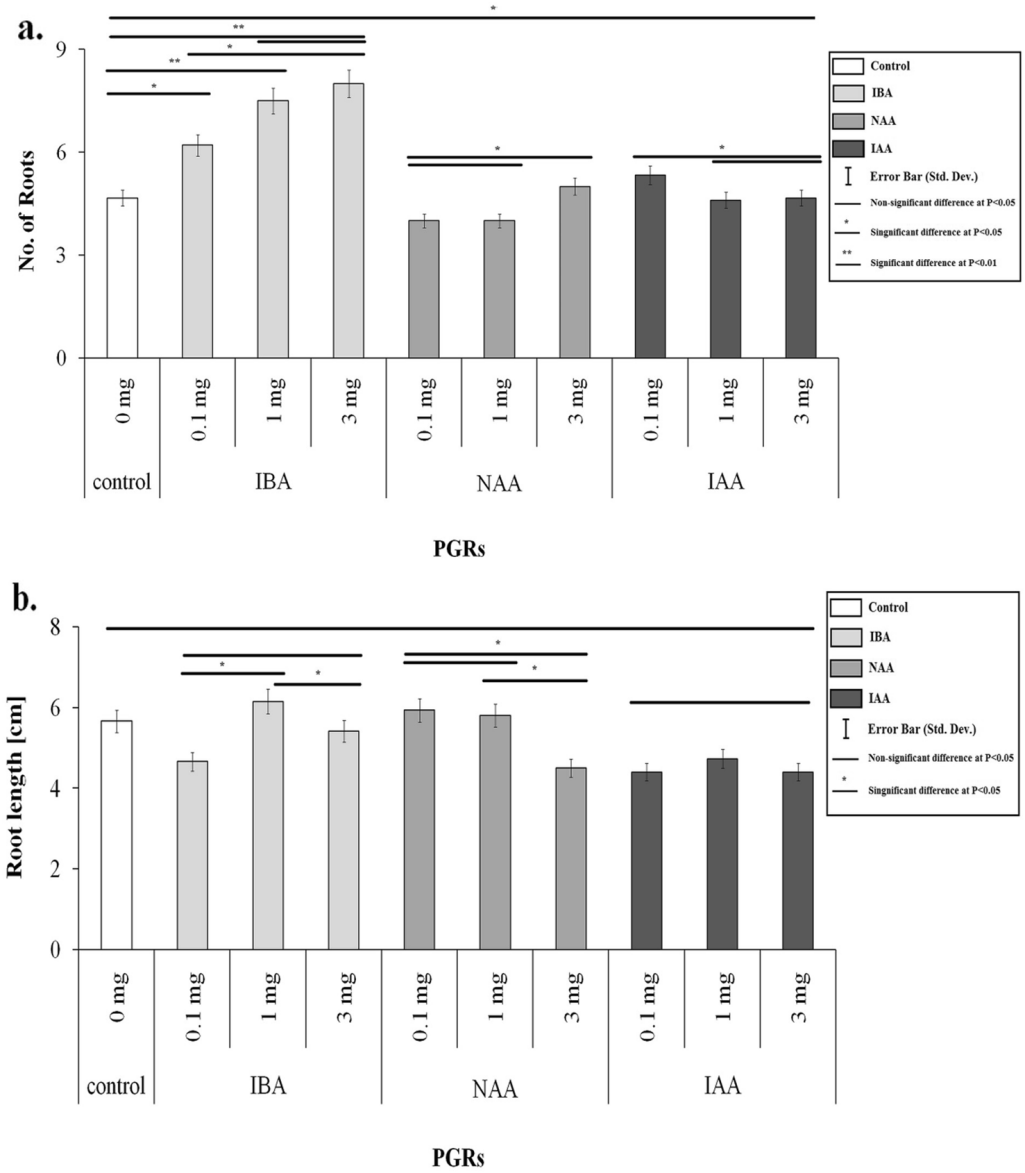
(**a**) Effect of PGRs on number of roots (**b**) Effect of PGRs on root length.

**Figure 3:**
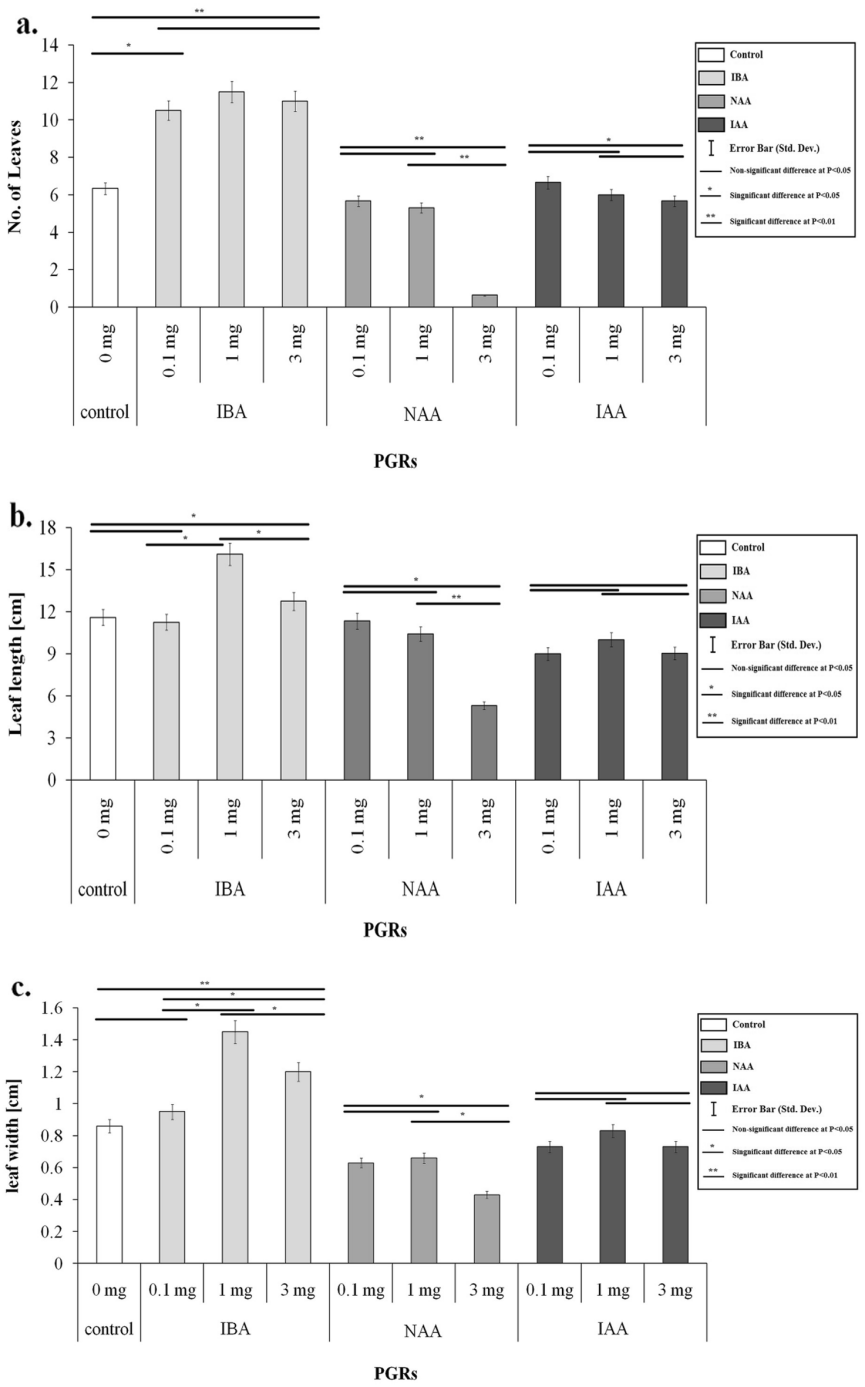
(**a**) Effect of PGRs on number of leaves (**b**) Effect of PGRs on leaf length (**c**) Effect of PGRs on leaf width.

**Figure 4:**
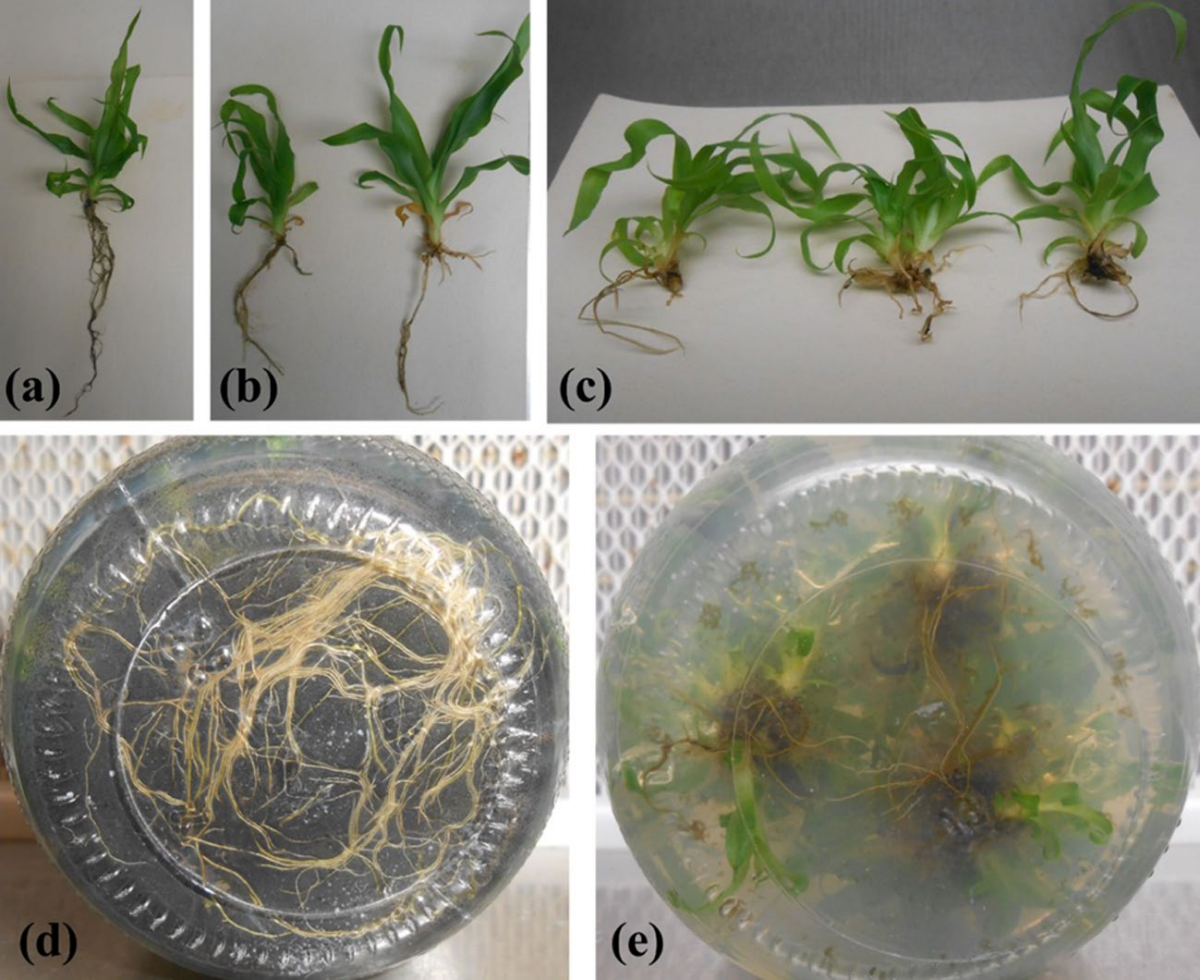
(**a**) Root and shoot growth on the medium containing 1.0 mg L^−1^ IBA, (**b**) Root and shoot growth on the medium containing 1.0 mg L^−1^ NAA, (**c**) Root and shoot growth on the medium containing 3.0 mg L^−1^ IBA, (**d**) Root formation in the plantlets on the medium containing 1.0 mg L^−1^ IBA (**e**) Root formation in the pineapple plantlets on the medium containing 1.0 mg L^−1^ NAA.

Adventitious rooting of in vitro plantlets is a crucial step influenced by different endogenous factors such as phytohormones and environmental factors. The PGRs have a strong influence on the success of in vitro culture^12^. The influence of various treatments of PGRs was studied on the root and shoot growth of pineapple plantlets (Figs. 2, 3). Use of different auxins and their levels in the medium exhibited significant differences in relation to root induction and root number per plantlet (Fig. 4a). It was noted that among the PGRs used, IBA (1.0 mg L^−1^) added in MS medium exhibited a significant effect on rooting in plantlets (Figs. 2, 4a).

The effect of PGRs on root number in in vitro plantlets showed that IBA significantly produced the highest root number (avg. 8) as compared to NAA and IAA (Figs. 2, 4a,b). The root number increased significantly by increasing the IBA concentration from 1.0 to 3.0 mg L^−1^ (Fig. 4c). However, the root number values did not show a significant difference (P > 0.05) using 1.0 mg L^−1^ and 3.0 mg L^−1^ in the medium. Still, they showed a significant difference in the values than other auxins added in the different media simultaneously. Moreover, non-significant differences were noted in various treatments of all auxin types regarding root length (Fig. 2b). Maximum root length (6.15 cm) was attained on the medium comprising of 1.0 mg L^−1^ IBA as compared to the control (5.66 cm) (Fig. 4d) and lowest in medium comprising 1.0 mg L^−1^ IAA (Fig. 4e). Each PGR concentration exhibited non-significant differences. Furthermore, the PGRs considerably impacted the number of leaves, leaf length, and leaf width. Further noted that different PGR levels affected the average leaf number in each plantlet. Leaf numbers in each plantlet varied on different IBA, NAA, and IAA treatments. The medium comprising 1.0 mg L^−1^ IBA exhibited a significantly highest average leaf number value (11.5) compared to NAA and IAA (Fig. 3a). In contrast, average leaf number values obtained on different treatments of IBA exhibited non-significant differences. NAA and IAA failed to exhibit any significant effects compared to the control. Increasing concentrations of NAA and IAA affected leaf growth negatively; for example, NAA at 3 mg L^−1^ produced a significantly lowest leaf number per plantlet (Fig. 3a). The detrimental effects of NAA and IAA levels on leaf length are depicted in Fig. 3b. The medium comprising of IBA (0.1–3 mg L^−1^) induced significantly highest average leaf length (P > 0.05) in plantlets than control and other PGRs treatments, with MS medium comprising of IBA (1.0 mg L^−1^) producing significant results, despite there being no statistically significant difference between the IBA at 0.1 mg L^−1^ concentration and control (P > 0.05) (16.1 cm). Similarly, Fig. 3c reveals that in plantlets treated with IBA (1.0 mg L^−1^ and 3 mg L^−1^), highest leaf length (1.2 and 1.45 cm, respectively) was obtained. In contrast, increasing the concentration of NAA had a significant negative effect (P < 0.05) noted on leaf width (Fig. 3c). IAA treatments had no influence (P > 0.05) on the leaf width.

Media containing NAA alone (1.0 mg L^−1^)^28^ and the addition of 2 mg L^−1^ IBA, 2 mg L^−1^ IAA^29^ induced roots in pineapple plantlets cv. Smooth Cayenne. The solidified half-level MS medium containing 0.5 mg L^−1^ IBA and 0.5 mg L^−1^ NAA^30^ noted better rooting. Medium comprising 0.5 mg L^−1^ IBA, 0.5 mg L^−1^ NAA^18^, and half-level MS medium containing 2.0 mg L^−1^ IBA^31^ recommended for rooting in cv. Primavita, MD2 and Madhupur cultivars.

Auxins played a crucial role in the growth of the stem, root, and leaf elongation. The results of the current investigation for in vitro rooting of pineapple on different PGR treatments revealed no significant variations (P > 0.05) in root number and length among the tested PGRs.

As exhibited in Fig. 2, adding IBA in the media increased significantly (P < 0.05) root number and length (Fig. 4d). Similar responses of IBA for inducing roots were described by Abul-Soad et al.^25^ and preferred to include NAA or IBA (1.0 mg L^−1^) in media to produce roots. Khan et al.^32^ and Devi et al.^33^ described that the best medium comprising only IBA induced significantly maximum roots per plantlet. The IBA is more effective in stimulating roots in in vitro plantlets due to the chemical content of IBA, which supports root formation^34,35^. According to Arlianti et al.^36^, IBA is frequently used for rooting compared to other auxin types. The role of IBA or NAA in enhancement of root length is well documented^18^. The findings of the positive effects of low NAA levels were most likely due to cultivar differences and endogenous plant auxin levels. NAA was applied for rooting in cv. Smooth Cayenne^15,28^.

Rooting in in vitro pineapple plantlets were also induced on the control medium (without PGRs) but exhibited significantly lowest in relation to root number, length and vigor as compared to media comprised of NAA or IBA. Moreover, the usage of auxins enhanced the number of leaves significantly (P < 0.05) (Fig. 3a) on the medium comprising 1 mg L^−1^ IBA. Similarly, Kiss et al.^37^ observed pineapple shoot multiplication on an IBA-containing medium. Aguiar et al.^38^ obtained significant leaf number in pineapple plantlets using IBA. NAA and IAA had no significant (P > 0.05) effects on the number of shoots. This result opposed the findings of Amany et al.^39^, who noted that NAA (0.1 mg L^−1^) resulted in the highest number of shoots/explant in Jackfruit. Solangi et al.^35^ observed a good root number and root length in in vitro date palm plantlets using 0.1 mg L^−1^ NAA and 0.1 mg L^−1^ BA. IAA and NAA levels did not affect leaf length (Fig. 3). Vesco^40^ achieved a similar result by improving the shoot length of pineapple plantlets on the medium devoid of PGRs. Jain and Haggman^41^ stated shoot elongation on the media without PGRs. Danso et al.^18^ described an optimistic impact on the increase in the length of shoots on the Auxins media.

### Effect of different soil mixtures on acclimatization of plantlets in greenhouse

**Figure 5:**
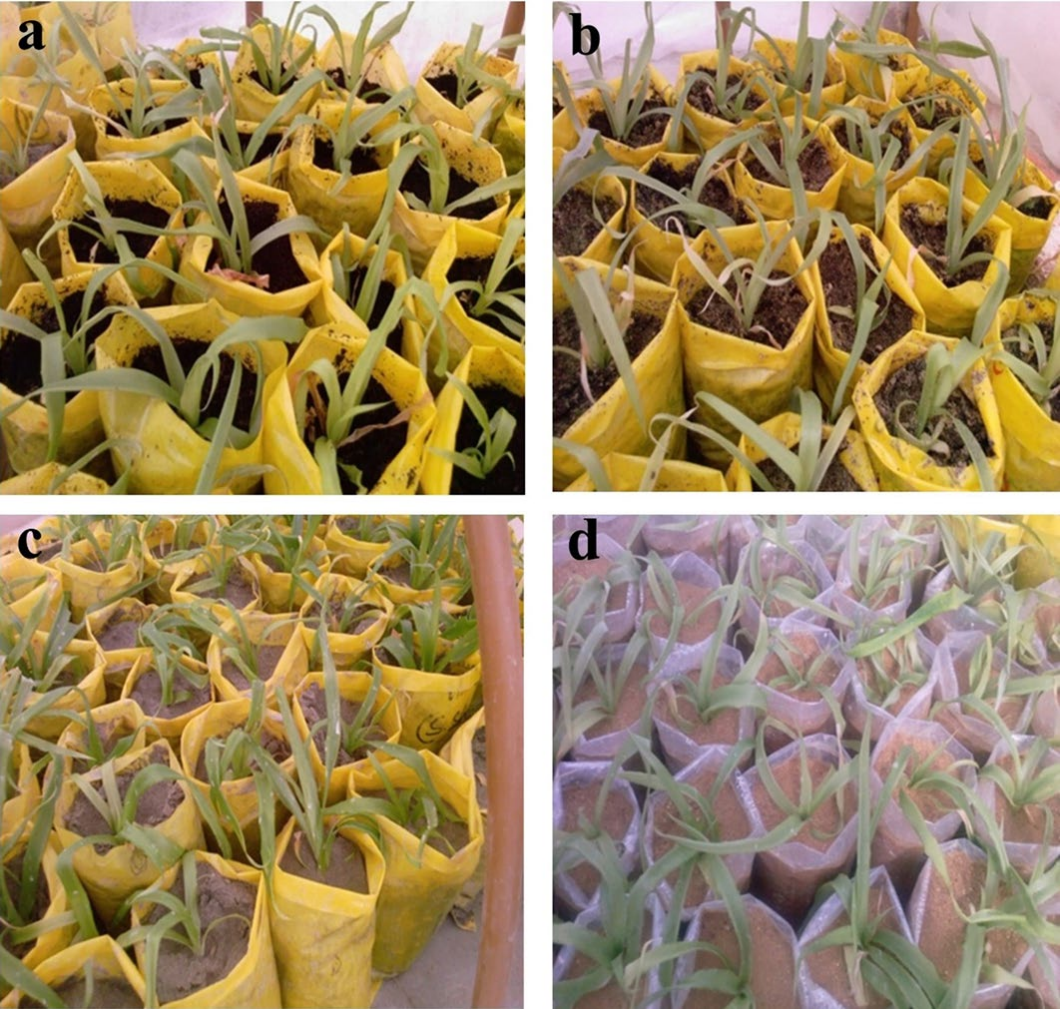
(**a**) Effect of peat moss on vegetative growth of plants in greenhouse (**b**) Effect of peat moss + bolhari (**c**) Effect of river silt (**d**) effect of bolhari.

**Figure 6:**
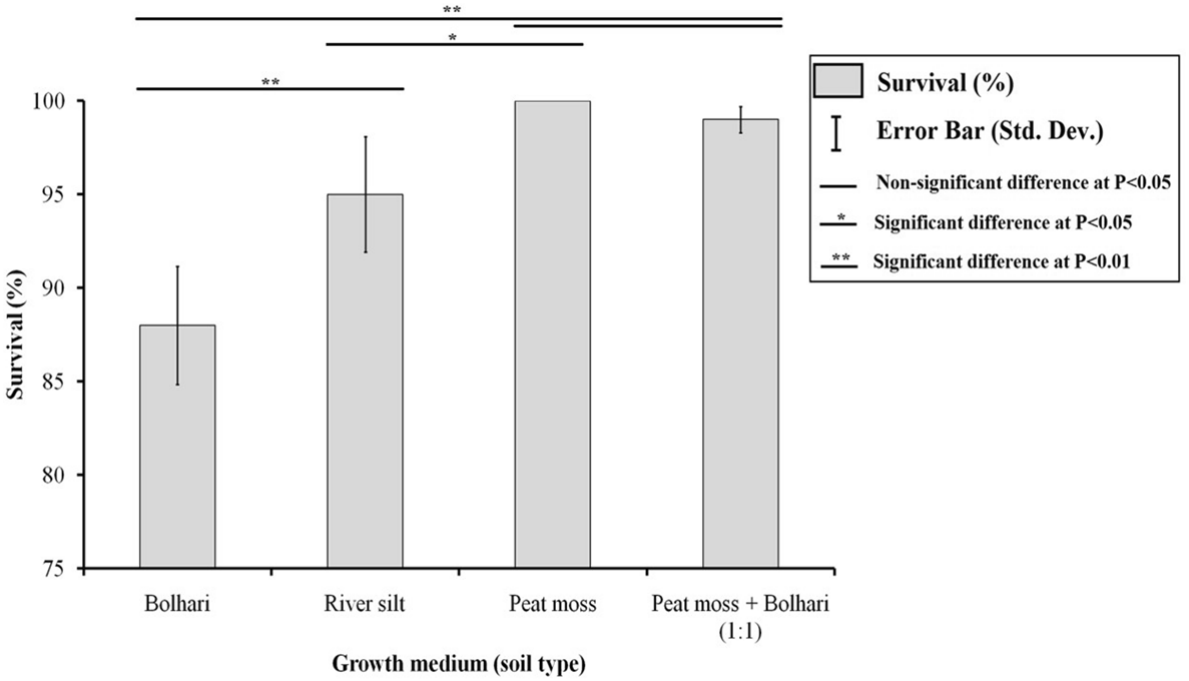
Effect of different soil types on ex vitro survival percentage of pineapple plantlets during acclimatization.

**Figure 7:**
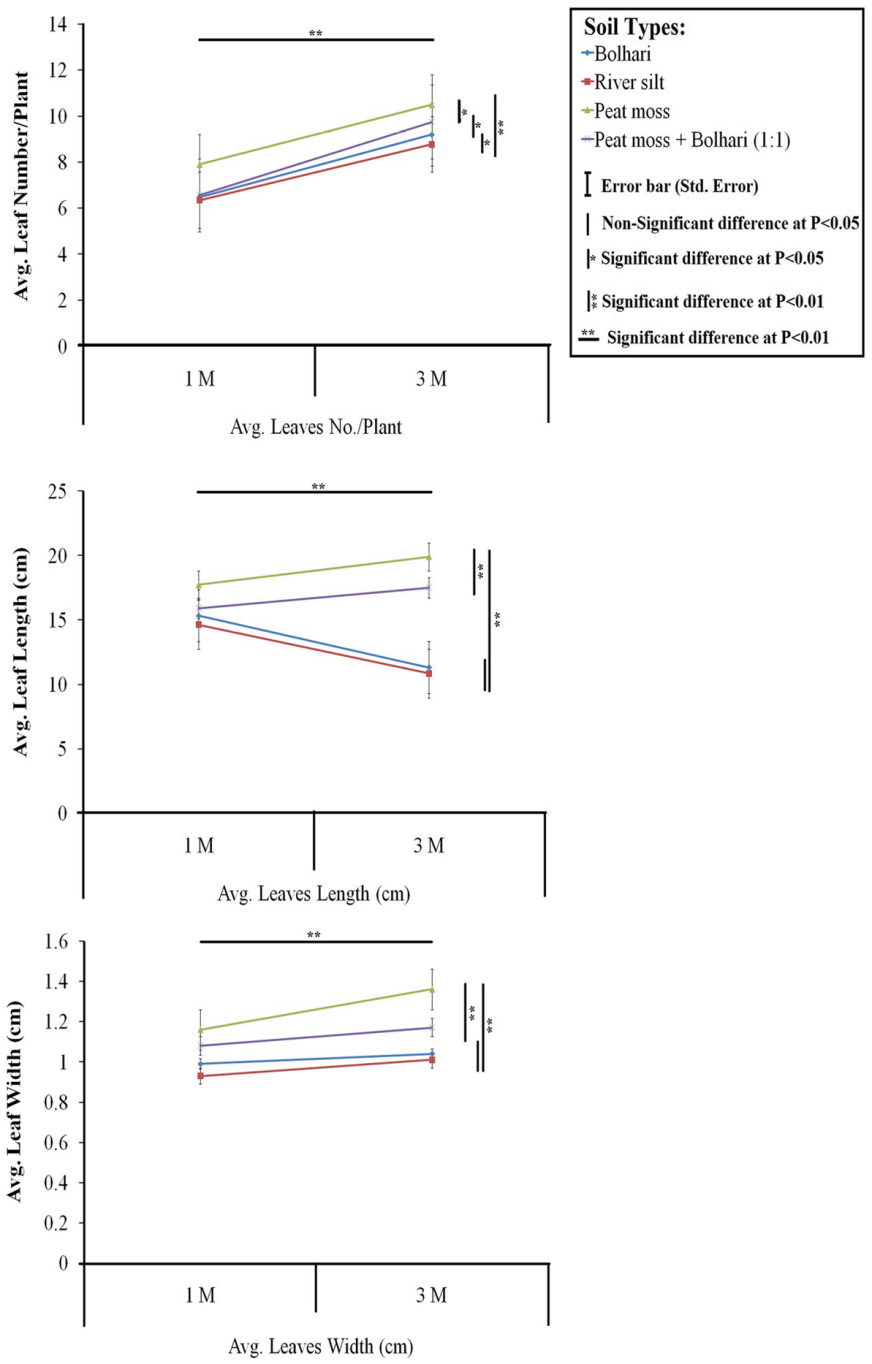
Effect of different soil types on ex vitro shooting of pineapple during acclimatization.

Well-rooted plantlets (Fig. 5a,b) were transferred in the different soil mixtures. The average survival percentage of plantlets after one month of transplanting is shown in Fig. 6, as well as the influence of different soil combinations on leaf number, leaf length, and leaf width (Fig. 7). Plantlets grown in pure peat moss shown 100% survivability and developed considerably more leaf number and leaf length (P < 0.01) than other treatments (Fig. 7). Peat moss alone exhibited a significantly highest survival rate (100%) of plantlets in the greenhouse than other soil mixtures. Moreover, it was noted that peat moss significantly increased plantlet growth and vigor and acquired rapid growth of plantlets as compared to other soil mixtures. Furthermore, the higher survival rate of plantlets (98.9%) transplanted on bolhari + peat moss (1:1) recorded while on river silt, the total survival rate of plantlets was 59.1% as significantly lowest. On bolhari soil medium, plantlets had a survival percentage of 86.9%. However, this was also significantly lowest (P < 0.01) compared to other kinds of soil media (Fig. 6). During the first week of transplanting, high humidity was crucial for the survival of plantlets, therefore all plantlets were covered with polyethene plastic sheets to maintain the humidity. The pineapple plants were shifted to larger plastic pots after three months of transplanting. Because of slow growth, pineapple plants require a long acclimatization period to reach a proper size^42^. Although humic acid and plant growth-promoting bacteria can be exploited to shorten the growth period during acclimatization^43^. Acclimatization success still needs to be improved by the type of growing soil media used. Soil media has proven to impact plant survival and growth in a greenhouse^44^.

Significantly highest growth values (leaf number and leaf length) were noted in the plantlets grown on a soil medium comprising only peat moss as compared to other treatments (Fig. 6). Another soil mixture containing bolhari + peat moss was the second best-growing media, according to the data, indicated a positive increase in leaf number, leaf length, and leaf width in the greenhouse but the results were non-significant statistically by comparing the results obtained using only peat moss alone and peat moss + bolhari (1:1) demonstrating the importance of bolhari. Hence, the soil medium type significantly impacted the growth of acclimatized plantlets. Peat moss appeared to be superior to other types of growing media for the adaptation and growth of plantlets in the greenhouse (Fig. 6). Atawia et al.^7^ reported that uniform growth of plants in the greenhouse strongly associated with the use of peat moss in the mixture. Abul-Soad et al.^25^ obtained the 100% survival of the pineapple plantlets on the soil medium containing only peat moss. The results were in accordance with the findings of Folliot and Marchal^45^, who discovered that peat moss performed better in the acclimatization of pineapple plantlets. The semi-composed pine bark improves the structure of the substrate, such as porosity, allowing proper gaseous exchange between the environment and substrate^46^. The current study also recommends using peat moss holding similar characteristics such as appropriate nutrient sources and providing proper gaseous exchange to roots. MD2 pineapple-rooted plantlets were acclimated in jiffy peat moss in the greenhouse, resulting in optimal growth^18^. The usage of artificial and commercial soils like peat moss is significantly connected to the increasing quantity and length of leaves, as well as optimal plantlet growth in vivo. This could be attributed to peat moss defining properties, which include standard composition, stability, water, and air holding ability, low pH and nutrients, and absence of weed seeds, insects, and pests.

Growth of aerial parts and survival percentage of plantlets were achieved using peat moss mixture and bolhari in jackfruit plants^39^, peat moss and perlite (1:1) in Amelanchier plantlets^47^, and grapevine plantlets^48^. At the same time, bolhari and river silt (1:1), rice husks and sand mixture were found suitable for pineapple plants^14,49^.

## Conclusions

Different treatments of auxins tested induced roots and leaves in pineapple plantlets during in vitro growth stage, which proved fruitful for better plantlets' growth in the greenhouse. Auxins (IBA and NAA) gave promising results in relation to producing healthy and long multiple roots, leaf number and leaf length and exhibited healthy growth in the greenhouse. The different soil mixtures were also tested for the growth of plantlets in the greenhouse. Better growth of plantlets was achieved on the soil medium comprising only peat moss. Cultivation of pineapple in the area is need of the time because it is one of the important fruits bearing crops which is the source of health-promoting nutrients. The significant results obtained during in vitro and ex vitro growth of plantlets can be applicable for growing other pineapple cultivars.

## Data Availability

The datasets generated during and/or analyzed during the current study are available from the corresponding author upon reasonable request.
